# How correct is the correct length for central venous catheter insertion

**DOI:** 10.4103/0972-5229.58543

**Published:** 2009

**Authors:** Rash Kujur, S. Manimala Rao, M. Mrinal

**Affiliations:** **From:** Department of Critical Care, Yashoda Hospital, Somajiguda, Hyderabad, India

**Keywords:** Carina, central venous catheters, right and left internal jugular vein

## Abstract

**Background and Aim:**

Central venous catheters (CVC) are important in the management of critically ill patients. Incorrect positioning may lead to many serious complications. Chest radiograph is a convenient means of determining the correct position of the catheter tip. The present study was designed to evaluate the depth of CVC placed through the right and left internal jugular vein (IJV) in order to achieve optimum placement of the catheter tip.

**Materials and Methods:**

A total of 107 patients in whom CVCs were put through either the right or left IJV through a central approach were included in this prospective study. Catheter tip position was observed in the post procedure chest radiograph. It was considered correct if the tip was just below the carina in the left-sided catheters and just above carina in the right-sided catheters. The catheters were repositioned based on the chest radiographs. The catheter depth leading to optimum tip placement was noted.

**Results:**

In males, catheter repositioning was required in 13 of 58 patients (22.41%) in the right IJV catheters, whereas in 2 of 13 patients (15.38%) in the left IJV catheters. In females, repositioning was required in 12 of 25 patients (48%) in the right IJV catheters and 2 of 11 patients (18.18%) in the left IJV catheters. Repositioning rate was higher in females (14/36) compared with males (15/71), which was statistically significant (*P* = 0.05, 95% CI). Repositioning rates were significantly higher in females (12/25) as compared with males (13/58) in the right IJV catheters (*P* = 0.019, 95% CI).

**Conclusion:**

By cannulating the IJV through a central approach, the catheters can be fixed at a length of 12-13 cm in males and 11-12 cm in females in the right IJV and at a length of 13-14 cm in males and 12-13 cm in females in the left IJV in order to achieve correct positioning.

## Introduction

Central venous catheters (CVC) play a very important role in the management of critically ill patients in both ICUs and special wards. Apart from thrombosis and infections, their use is associated with many mechanical complications such as cardiac tamponade, perforation, pneumothorax and life-threatening arrhythmias.[1,2]

For proper monitoring of central venous pressure and to avoid some of the life-threatening mechanical complications it is mandatory that the tip of the catheter lies in the correct position. The correct position of the tip of CVC is considered to be in the superior vena cava (SVC) above the level of pericardial reflection.[3,4] It is also recommended that the catheter tip should lie in the long axis of the SVC, without acute abutment to the vein wall.[4] Various methods such as anatomical landmarks,[5,6] simple formulae,[7] right atrial electrocardiography[8–10] and echocardiography[11] have been used to ensure correct placement of the CVC tip.

In this study, we evaluated 107 patients in whom CVCs were placed as part of the management. The position of the tip of the catheter and the rate of repositioning was observed. The position of the CVC tip was considered correct in the chest radiograph if it was just above the carina in right-sided catheters and just below the carina in left-sided catheters.

## Materials and Methods

After approval by the institutional ethics committee, 107 patients in the age group of 20-60 years were enrolled in this prospective observational study. CVCs placed through the right and left internal jugular veins (IJVs) were evaluated. Patients with altered coagulation parameters, arrhythmias, pacemaker *in situ*, short neck, burn contractures of the neck and cervical spine injury were excluded from the study. All catheters were placed by intensivists and trained doctors.

All catheters (double/triple lumen Certofix B Braun) were placed blindly through the central approach using the Seldinger's technique.

The catheters were inserted to a length of 13-14 cm in right-sided catheters and to a length of 14-15 cm in left-sided catheters. A post procedure portable chest radiograph was performed in all the cases in supine position, for assessing the position of the tip of the catheter. CVC tip position was judged as correct if the tip was positioned just above the level of carina for right IJV catheters and just below the level of carina for left IJV catheters. Catheters were repositioned after checking the tip position in the chest radiograph. The depth of insertion of the catheter after repositioning was noted and was confirmed by a repeat chest radiograph.

## Results

Of the 107 patients evaluated, there were 71 male and 36 female patients. Total number of right IJV catheters was 83 (77.57%) and that of left IJV catheters was 24 (22.43%).

Among the 71 male patients, 58 (81.69%) had right IJV catheters, whereas 13 (18.3%) had left IJV catheters. Among 36 females, 25 (69.44%) had right IJV catheters, whereas 11 (30.55%) had left IJV catheters.

After observing the position of the catheter tip in post procedure chest radiograph, catheter repositioning was required in 15 of 71 males and 14 of 36 females. Chi-square test performed showed that repositioning rate was higher in females which was statistically significant (*P* = 0.05, CI 95%). Repositioning rate in right-sided IJV catheters (25/83) compared with left-sided ones (4/24) was not significant (*P* = 0.19, CI 95%). Repositioning was done in 13 of 58 (22.41%) patients in right-sided IJV catheters, whereas in 2 of 13 (15.38%) in left-sided IJV catheters in males, whereas repositioning was required in 12 of 25 (48%) in right-sided IJV catheters and in 2 of 11 (18.18%) in left-sided IJV catheters in females. Repositioning rates were significantly higher in females (12/25) as compared with males (13/58) in right-sided IJV catheters (*P* = 0.019, CI 95%), whereas no significant difference was observed in males and females with regard to left-sided IJV catheters.

After repositioning, chest radiograph was repeated to confirm the position of the tip and the catheter insertion depth was noted.

## Discussion

CVC insertion is associated with many mechanical complications with an incidence varying from 6.2% to 11.8% in subclavian and internal jugular approaches.[2] These include cardiac tamponade, cardiac perforation, vessel wall as well as cardiac erosions, tricuspid valve damage, malignant atrial and ventricular arrhythmias, pneumothorax and hemothorax.[1,2,7,8,12] These complications result from erosion through vessels and atrial or ventricular chambers by the catheter tip.[3,4,7]

Moreover, CVCs not inserted to an adequate depth could easily lie outside the SVC, potentially increasing the risk of thrombus formation and/or infection.[3]

Vessel wall and cardiac perforation can occur immediately during the procedure either by guidewire, dilator or over insertion of the catheter. It can occur late as well, as either secondary to catheter advancement with head, arm and trunk movement or by tissue erosion caused by catheter tip abutting against vessel or cardiac wall, which is further aggravated by cardiac contractions.[3,4,7] It has also been observed that left-sided catheter placement was associated with a higher risk for vascular erosion.[7]

Over insertion of the catheter can also lead to various arrhythmias including atrial and ventricular premature beats, ventricular tachycardia or fibrillation. These rhythm disturbances are usually resistant to drug suppression and require withdrawal of the catheter from the cardiac chambers.[1,7]

McGee *et al*., observed that, using 30-cm catheters, 47% of their catheter tips were located in the heart chambers and concluded that by using a 15- or 16-cm central venous catheter, cardiac cannulation may be reduced.[13]

Several recommendations have been made to decrease the risk of vessel or cardiac perforation, which include omission of beveled or hard tip catheter, avoidance of left-sided approach and immobilization of the catheter.[7] But the most effective method found was to place the tip in an extracardiac position in SVC and confirming the placement by chest radiograph.[3,5,7]

According to the recommended guidelines CVC tip should lie in the SVC above the pericardial reflection to prevent such potential and serious complications.[6,8,14] It is also recommended that the catheter tip should lie in the long axis of the SVC without acute abutment to the vein wall.[3,4] It has been shown in the laboratory that an angle of the CVC tip to vessel wall of greater than 40° is more likely to lead to vessel wall perforation.[4]

**Figure 1:**
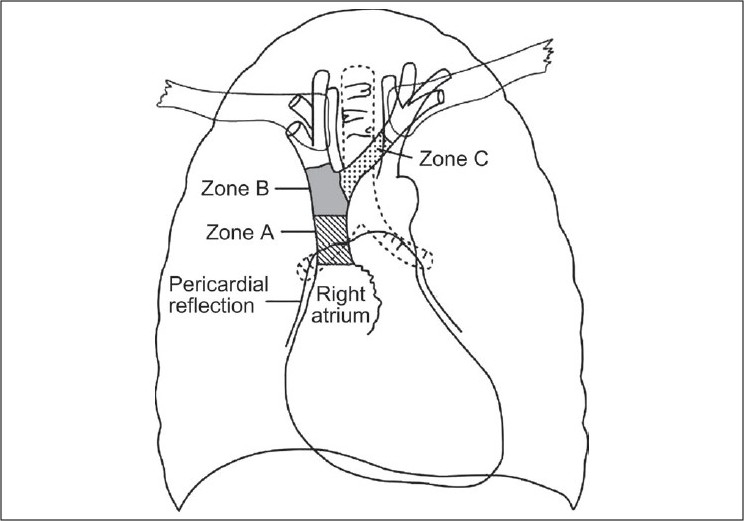
Stylized anatomical Figure dividing the great veins and upper RA into three zones (A-C), representing different areas of significance for placement of CVCs

Schematic zones for catheter tip positioning can be categorized into three zones [Figure 1].[4] Zone A represents the lower SVC and upper RA. In this zone CVCs placed from the left side are likely to lie parallel to the vessel walls. This may represent a necessary compromise for left-sided CVCs to ensure that they lie parallel to the vessel wall. Right-sided CVCs in this zone, however, should be pulled back to zone B. Zone B represents the area around the junction of the left and right innominate veins and the upper SVC. This is a suitable area for CVCs placed from the right side; however, left-sided CVCs will enter this area at a steep angle and are at risk of abutting the lateral wall of the SVC and should ideally be advanced into zone A. Zone C represents the left innominate vein proximal to the SVC. CVCs in zone C are probably suitable for short-term fluid therapy and CVP monitoring, but not for inotrope infusions or long-term use. The safety of this site has been questioned.[4]

The upper limit of the pericardial reflection cannot be seen on plain chest X-ray but is generally accepted to be approximately 0.8 cm below the carina.[14] This has been assessed in preserved and fresh cadavers.[4,14] Moreover, it is easily visible even in a poor quality portable anteroposterior chest radiograph. Hence, carina has been considered as a radiological landmark for CVC tip position.[14,15] Zones A and B mentioned in the diagram are in relation to the carina.

Site of insertion, patient's height and body habitus are significant factors that influence the appropriate catheter insertion length.[7] Several methods have been adapted to safely place the catheter tip in the desired place in the SVC. Right atrial electrocardiography has been used to guide the catheter tip to the suitable position but it requires special gadgets, which may not be readily available in certain emergency situations.[7,9,10]

In a study, Peres utilized patients' height to develop formulas to predict the optimum length of the catheter to be inserted for right internal or external jugular catheters, right infraclavicular subclavian catheters and left external jugular catheters.[16]

But Peres' formula does not take into account the probable differences in catheter insertion length due to variation in same side approach, that is, high vs cricoid internal jugular approaches or medial vs lateral subclavian approaches.[7]

Moreover, in certain emergency situations time and circumstances may not permit rough measurement of the patient's height. In such situations average catheter insertion length calculated for each site may be used for correct placement of the catheter tip and avoid unnecessary complications.[7] In a prospective observational study of 127 patients, McGee *et al*., suggested an insertion depth of 16 centimeter to be safe in internal jugular and subclavian route.[17] Russel *et al*., suggested a length of 13 cm for the CVCs to be appropriate.[18]

## Conclusion

In our study, we concluded that post procedure chest radiograph is helpful in evaluating the position of the tip of the catheter in relation to the carina. While inserting the CVC in the IJV via the central approach, the depth of insertion could be at 12-13 cm in males and 11-12 cm in females in right-sided catheters, whereas at a depth of 13-14 cm in males and 12-13 cm in females in left-sided ones. At this length the catheter tip could lie in an optimum position. In this way number 13 may not be lucky for all. A depth of 16 cm may be adequate for western population, but Indian population may require shorter length of placement.
